# Live cell flattening — traditional and novel approaches

**DOI:** 10.1186/1757-5036-3-9

**Published:** 2010-04-19

**Authors:** Christian Westendorf, Albert J Bae, Christoph Erlenkamper, Edouard Galland, Carl Franck, Eberhard Bodenschatz, Carsten Beta

**Affiliations:** 1 Max-Planck-Institut für Dynamik und Selbstorganisation, 37077 Göttingen, Germany; 2 Laboratory of Atomic and Solid-State Physics, Cornell University, Ithaca, NY 14853, USA; 3 Universität des Saarlandes, 66123 Saarbrücken, Germany; 4 Ecole Polytechnique, 91128 Palaiseau Cedex, France; 5 Institut für Nichtlineare Dynamik, Georg-August-Universität Göttingen, 37073 Göttingen, Germany; 6 Institut für Physik und Astronomie, Universität Potsdam, 14476 Potsdam, Germany

## Abstract

Eukaryotic cell flattening is valuable for improving microscopic observations, ranging from bright field (BF) to total internal reflection fluorescence (TIRF) microscopy. Fundamental processes, such as mitosis and *in vivo *actin polymerization, have been investigated using these techniques. Here, we review the well known agar overlayer protocol and the oil overlay method. In addition, we present more elaborate microfluidics-based techniques that provide us with a greater level of control. We demonstrate these techniques on the social amoebae *Dictyostelium discoideum*, comparing the advantages and disadvantages of each method.

**PACS Codes:** 87.64.-t, 47.61.-k, 87.80.Ek

## 1. Background

Studies of biochemical reactions are traditionally carried out on large cell populations, typically on the order of a million cells or more. Based on the biochemical data from such studies, systems biology has greatly advanced our knowledge of metabolic pathways and signaling cascades in living cells [1]. Yet, as our understanding of biochemical interaction networks becomes more complete, it is advantageous to complement this progress with fluorescence microscopy experiments that characterize the dynamics of such pathways on the single cell level. With fluorescence microscopy, we can observe the expression levels and localization of tagged proteins in each cell. One also can examine the properties of and interactions between proteins using Fluorescence Correlation Spectroscopy and Förster Resonance Energy Transfer.

Some of the limitations in live cell microscopy are due to the movement of cells in and out of the plane of focus. To overcome these limitations, cells can be flattened to quasi two-dimensional geometries, ensuring that the cells remain in the focal plane. These flattened geometries have been advantageous for observing the eukaryotic cytoskeleton during chemotaxis and cytokinesis [2,3]. Recent examples combined cell flattening with Total Internal Reflection Fluorescence (TIRF) microscopy to increase the area of observation and keep the cell membrane and cortex in the evanescent field [4].

In this article, we present a brief review of established overlay approaches for cell flattening. Furthermore, we introduce microfluidic techniques to achieve greater control over the degree of flattening. Each technique is exemplified with a short demonstration using cells of the social amoeba *Dictyostelium discoideum *[5]. We discuss the advantages and disadvantages of each flattening technique. Our aim is to provide a reference for readers from the field of live cell imaging, who require advice in choosing a cell flattening system.

## 2. Methods

### 2.1. Cell culture

Experiments were carried out using the AX2 derived, LimE-GFP tagged strain of *D. discoideum *[6] and the RI9 derived, cAR1-GFP strain [7]. Cells were cultivated in HL-5 medium (ForMedium) at 22°C on polystyrene dishes (Primaria, Falcon), or shaken in suspension at 150 rpm. Cultivation of the cAR1-GFP mutant required addition of 5 *μ*g/ml G418 (Geneticin, Gibco) to the HL-5 medium. To examine chemotactic cells, the culture was starved in a 20 ml phosphate buffer (PB, pH = 6) shaking cell suspension, prior to each experiment [8]. This suspension was supplied with 60 *μ*l of 18 *μ*M cAMP (Sigma) every 6 minutes to synchronize the cells [9]. The developed cells were harvested by washing the suspension three times in PB and then used in the experiments discussed below.

### 2.2. Microscopy

Bright field (BF) and TIRF microscopy were conducted on an Olympus IX81 setup, controlled with the software Cell^R^. Observations of *D. discoideum *were carried out using a 60× PlanApo N oil immersion or a 20× LUCPlan FLN objective. The GFP linked to the DdLimE and cAR1 respectively was excited by the 488 nm line of a 20 mW Ar laser. BF and TIRF images were taken with a 16 bit, 1344 × 1024 pixel CCD camera (Hamamatsu Photonics). MATLAB (MathWorks) was used for data analysis. Brightness and contrast were adjusted using ImageJ [10]. Confocal laser scanning microscopy was conducted on the Olympus Fluoview1000 setup, using a 60× UPlanSApo objective. Excitation of the GFP was provided by the 488 nm line of a 150 mW Ar laser. All flattening techniques have been characterized by taking a confocal z-stack, using cAR1-GFP cells. Since cAR1 is a membrane bound G-protein coupled receptor, cAR1-GFP marks the membrane of the *D. discoideum *cell. The confocal z-stacks were depth corrected as previously described [11].

### 2.3. Agar overlay

In the agar overlay method, the cell suspension is sandwiched between a layer of glass below and a sheet of agarose above. As fluid is removed from between these layers, the agar overlayer is lowered, and presses on the cells. A detailed description of the agar overlay method can be found in [12,13]. Approximately 200 *μ*l of a *D. discoideum *suspension in PB was placed on a cover slip (24 × 60 mm, No. 1, Menzel Gläser). An agar sheet (4% agarose, UltraPure, Invitrogen) of *ca*. 0.15 mm thickness was placed on top of the cell suspension. Cell flattening was induced by removal of PB with filter paper (Nr. 2, Whatman International). The degree of flattening could be controlled by removing different amounts of PB.

### 2.4. Oil overlay

**Figure 1:**
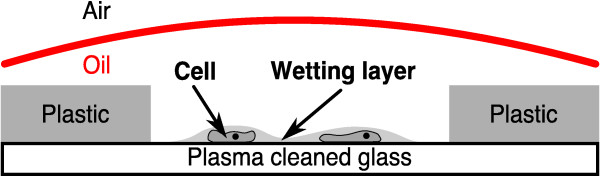
**Oil overlay technique**. A drawing of the oil overlayer technique on a modified glass bottom Petri dish. The arrows mark the *D. discoideum *cells (not to scale) and the surrounding wetting layer. Most of the wetting fluid is removed by a cotton thread (not shown) placed on the glass surface, which leads to flattening of the cells.

The second flattening approach is based on an oil overlay, instead of an agar sheet. Similar to the agar overlay, the underlying principles of this method have long been established. For an early reference from the *Dictyostelium *literature see [14]. Here, we proceeded as follows:

An amount of 100-200 *μ*l of a *D. discoideum *cell suspension is placed on a glass bottom Petri dish (Matek). The dish is 50 mm in diameter, with a 14 mm circular well in the center. To maximize the hydrophilicity of the surface, we replaced the glass bottom with a fresh cover slip (No. 1, Menzel Gläser), and this modified dish was then plasma cleaned for ~30 seconds (PDC 002, Harrick Plasma) immediately before depositing the cells. After waiting 15 minutes, to allow the cells to settle, we removed most of the PB with a pipette, leaving a thin wetting layer on the glass surface. A wet cotton thread is then laid along the circumference of the well. To prevent the wetting layer from drying out, we coat it with an oil layer (Neutral oil, Euro OTC Pharma), leaving one end of the thread exposed to the air outside. As this portion of the thread dries, the aqueous fluid underneath the oil layer is gently wicked away, resulting in a thinning of the wetting layer. When the desired degree of flattening is achieved, the wick is removed. The setup is illustrated in Figure 1.

### 2.5. Microfabrication and device characterization

We used standard PDMS-based soft-lithography to fabricate microfluidic devices [15]. A chrome mask (Cornell Nanofabrication Facility, Cornell University) was used to create a photoresist micro-pattern (SU8-25 or SU8-50, Microresist Chem) on a Si-wafer (polished, diameter 100 mm, SiMat) by photolithography. See the individual sections below for the different mask geometries. The height of the SU-8 pattern was measured with a white light interferometer (Wyko NT 1100, Veeco). Polydimethylsiloxane (PDMS, Sylgard 184, Dow Corning) was molded against the patterned master wafer. Air bubbles were removed in a vacuum chamber and the PDMS was then cured for 2 hours at 75°C. A block of PDMS carrying the imprint of the microstructure was cut out using a sharp knife. Inlets and outlets were punched into the PDMS with a clean syringe tip (19 gauge stainless steel, McMaster). The microstructured PDMS surface as well as the surface of the cover slip (24 × 60 mm, No. 1, Menzel Gläser) were treated by an air plasma for 5 minutes. Afterwards, bonding between the microstructured PDMS surface and the cover slip was initiated by bringing the two surfaces into contact with each other.

The microfluidic cell flattening devices described below commonly rely on changes in the height of the flattening chamber by deformation of the PDMS ceiling. Prior to a cell flattening experiment, a suspension of *D. discoideum *cells was filled into the microchannel through PTFE tubing (Novodirekt) via a 23 gauge sterile and sharpened syringe (Kruuse). The device was left for 15 minutes to allow the cells to settle down on the glass surface.

To quantify the changes in channel height, a 100 *μ*M solution of fluorescein (Acros Organics) in PB was filled into the channel, and a constant flow of 5 *μ*l/h was applied to prevent photobleaching [16]. The fluorescence was excited with a 150 W Xe/Hg mixed gas arc burner (part of the MT20 system, Olympus) and observed with a 10× Plan N objective. Our imaging region was large enough to cover the middle of the channel, as well as a portion outside of the channel (to get *I_back_*, used for background subtraction). We first measured the fluorescence intensity in the non-deformed flattening chamber and associate the corresponding intensity value *I*(0) with the maximal channel height *h_0_*, measured as described above. Assuming that the intensity *I *scales linear with the height *h*, we estimated the height of the flattened chamber by(1)

 is averaged over at least 100 pixels and the corresponding standard deviation in *I *is used for calculating the error bars.

### 2.6. Single layer microfluidic device

**Figure 2:**
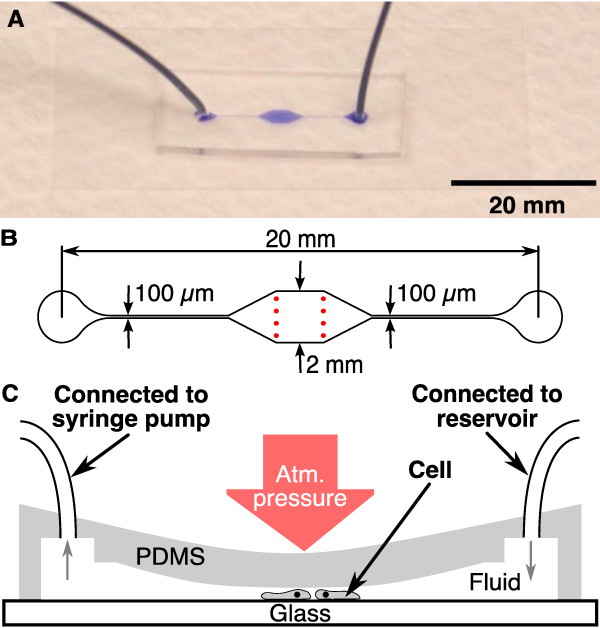
**Single layer microfluidic device**. (A) Assembled single layer device with connected tubing, filled with ink. (B) Geometry of the chrome mask used to create the single layer device. The height of this channel was 14 *μ*m. Two rows of pillars (red spots) support the ceiling. In (C), the principle of operation is shown. The inlet of the collapsing device is connected to a reservoir, filled with PB. A syringe pump removes the fluid at a constant rate (indicated by the gray arrows) and thus decreases the pressure within the microfluidic channel. This leads to a collapse of the ceiling and therefore to flattening of *D. discoideum *cells located in the channel.

The single layer microfluidic device consists of a single PDMS layer with a simple through-flow micro-channel, see Figure 2A. The key feature of this channel is the high aspect ratio (width ≫ height) chamber in the middle section. A syringe pump is used to withdraw liquid through the channel. The higher the flow rate, the greater the pressure drop between the outer atmosphere and the chamber. This pressure difference lowers the elastic PDMS ceiling, causing the cells below to be flattened.

Figure 2B shows the layout of the chrome mask used to fabricate the single layer microfluidic device. The final height of the microfluidic channel after spincoating the SU8-25 photoresist was 14 *μ*m (the taller the microfluidic channel, the higher the flow speed required to flatten cells inside the channel). The area of the chamber, where experiments were carried out was 2 × 2 mm in size. It was stabilized by two rows of pillars (Figure 2B, in red), to prevent uncontrolled collapsing. We used 45 g of PDMS and 4.5 g of curing agent (100:10), to create a *ca*. 3 mm tall microfluidic device. After the device was assembled, it was heated at 75°C for 10 minutes to prevent an untimely, irreversible collapse of the chamber ceiling. The cells were loaded as described above. Then, the outlet was connected to a reservoir of PB (see Figure 2C). To lower the PDMS ceiling, PB was withdrawn from the channel at a constant rate using a syringe pump (PHD 2000, Harvard Apparatus). The degree of flattening depends on the suction rate. It is crucial to remove bubbles from the microchannel, the tubing, and the syringes, since they may expand in volume, leading to an uncontrolled drop in the actual flow speed in the microchannel.

### 2.7. Microfluidic closed-end actuator

**Figure 3:**
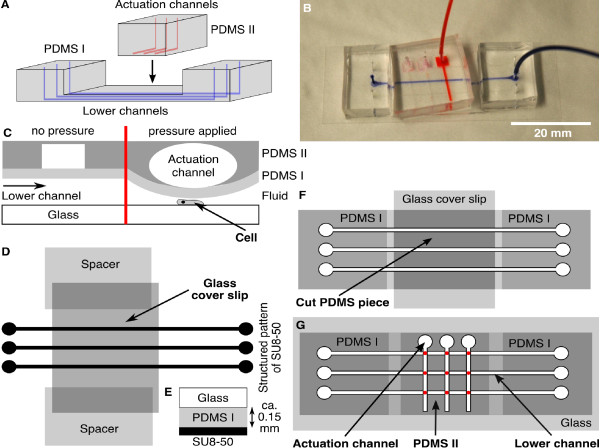
**Microfluidic closed-end actuator**. (A) The closed-end actuator consists of a lower layer (PDMS I) with channels containing the cells (blue) and an upper layer (PDMS II) with the actuation channels (red). (B) Photograph of the assembled closed-end actuator filled with ink (actuation channel in red and lower channel in blue). (C) The principle of operation for the closed-end actuator. (D) To create the lower layer, PDMS was poured onto the wafer. Then a cover slip was placed across the pattern. This cover slip rests on two other cover slips, which act as spacers that define the height of the PDMS layer (E). This layer will separate the lower and the upper channels. (F) After curing and cutting the PDMS, the middle section was cut out and stored. The first cover slip was replaced by a second cover slip and the cut PDMS piece is placed in its former position to provide stability during plasma cleaning. (G) Illustration of the assembled double layer device. The lower layer (PDMS I) contains the microfluidic channel filled with the cells. The upper layer (PDMS II) contains the closed-end channel which applies pressure to the channel below. The red spots in (G) mark the overlapping areas, in which flattening of the cells occur.

The closed-end actuator for microfluidic cell flattening was inspired by the PDMS-based fabrication of microfluidic valves introduced in [17] and relies on the same principle as the device described in [16]. This microfluidic device consists of two perpendicularly oriented arrays of channels in two PDMS layers, see Figure 3A and 3B. The channels of the lower layer contain the cells that are to be flattened. The actuation channels in the top layer are closed-end channels, i.e., they contain an inlet but no outlet. When pressure is applied to the inlet of an actuation channel, the thin PDMS film separating the upper and lower layers deforms, causing the height of the lower channels to decrease (Figure 3C).

The channels in the lower layer are 3 cm in length, 15 *μ*m in height and 500 *μ*m in width (Figure 3, PDMS I). This lower layer is made of a 100:9 mixture of PDMS to curing agent. Two cover slips (No. 1) are placed in the PDMS, on opposing sides of the structured pattern. These cover slips act as spacers, fixing the height of a third cover slip (24 × 60 mm, No. 1) placed across the microstructure (Figure 3D and 3E). After the PDMS cured, a rectangular block, containing the three channels is cut out. In this process, we must cut through the upper cover slip, so that the block consists of PDMS and the midsection of the upper cover slip. The ends of this cover glass, along with the spacers are left behind.

We take this block, and punch the inlet and outlet holes. Then, we carefully cut out the layer of PDMS above the embedded cover slip. It is critical that the corresponding cuts do not penetrate beyond the glass into the PDMS below; otherwise the channels will be damaged. The piece of PDMS, which was above the embedded cover slip, is put aside for use later. We remove all pieces of the cover glass from the main PDMS block. A fresh cover slip (22 × 40 mm, No. 1, Gold Seal) is placed on top of the thin midsection. The previously removed PDMS piece is replaced above the fresh glass (Figure 3F) and provides stability to the main block as it is plasma cleaned and bonded to a cover slip (24 × 60 mm, No. 1, Menzel Gläser). Finally, the stabilizing PDMS piece and cover glass are carefully removed from the block, and discarded. The lower layer is now assembled (PDMS I in Figure 3G).

The upper actuation channels are 1 cm in length, 30 *μ*m in height and 500 *μ*m in width (Figure 3, PDMS II). These closed-end channels are made from a 100:11 mixture of PDMS and curing agent. After curing, we cut out the PDMS block, and punch the inlets. We then plasma clean this upper layer with the previously assembled lower layer. We assemble the two layers such that the patterned side of the top layer (PDMS II in Figure 3G) is bonded on top of the mid-section of the bottom layer. The channels in the two layers are oriented perpendicular to each other. This concludes the construction of the closed-end actuation device.

We load the cells into the lower channel. After we allow the cells to settle and adhere to the glass substrate, we run a 5 *μ*l/h flow of PB. The cells located in the region of overlap between the lower and upper channels (Figure 3G, marked in red) are the cells that can be flattened.

Next, to apply pressure to the upper channels, we use an infuse/withdraw syringe pump to drive a 500 *μ*l syringe (Hamilton). The syringe and tubing are filled with water. The syringe pump is intrinsically a volume controlling device, not a pressure controlling device. We convert the volume change, induced by the syringe pump, into a pressure change by introducing a large air bubble (V_0 _≈ 200 *μ*l) near the plunger end of the syringe. Note that this bubble provides most of the compliance in the system, so Boyle's law tells us(2)

where *P*_actuation _is the pressure in the actuation chamber, and *V*_pump _is the volume injected by the syringe pump. By stopping the syringe pump, we can maintain a fixed pressure. To decrease the pressure, we simply run the syringe pump in reverse.

Note that, initially, the actuation channel is filled with air, but as pressure is applied, this air slowly permeates into the PDMS, and eventually will be displaced by water. This volume is negligible, when compared to *V*_0_, so the pressure equation above remains valid.

### 2.8. Microfluidic through-flow actuator

**Figure 4:**
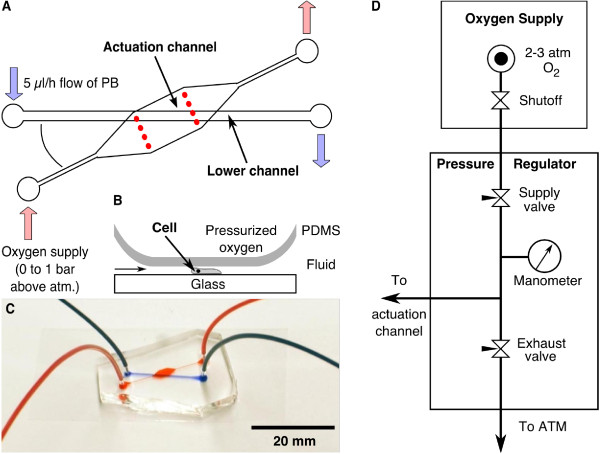
**Microfluidic through-flow actuator**. (A) Similar to the closed-end actuator, the cells are contained in the bottom channel. In this case, however, the actuation channel was open ended allowing oxygen to flow through. The oxygen was supplied to the cells via the permeable PDMS membrane which separates the two layers (B). By adjusting the pressure of the oxygen supply at the inlet, this PDMS membrane was deformed and flattened the cells. The angle between both channels was limited by the size of the cover slip. (C) Assembled double layer microfluidic channel with connected tubing. The actuation channel is filled with red and the lower channel with blue dye. (D) Schematic of the pressure regulation of the oxygen flowing through the actuation channel. The manometer measures the pressure applied to the actuation channel.

Similar to the previous device, the through-flow actuator is composed of two layers, an actuation channel on top and a channel for cell flattening below. The lower channel is filled with a *D. discoideum *cell suspension or a fluorescein solution for characterization. In contrast to the previous setup, the actuation channel is a through-flow channel connected to a continuous oxygen supply. The flow of oxygen is controlled by a metering valve. When the pressure in the actuation channel is increased, the PDMS sheet between the two layers is deformed, flattening the cells (see Figure 4B).

The microstructures were prepared by photolithography on two different master wafers. The layout of the first wafer, used for molding the actuation channel, was the same as for the single layer device (see Figure 2A) with a height of 28 *μ*m. The actuation channel was made by covering the wafer with a layer of the PDMS (100:10) about 1 cm thick. To prepare the thin lower layer, we used a second wafer with a channel structure of 500 *μ*m width and 24 *μ*m height (Figure 4A). In order to distribute the PDMS uniformly, two spacers were used, each consisting of three cover slips (No. 1) that had been glued together. The spacers were taped to opposing edges of the wafer and a bubble-free PDMS (100:10) droplet was placed between them. The long edge of a thick glass slide was used to evenly spread the PDMS between the two spacers. Before curing the PDMS, the spacers were removed.

The actuation channel was cut out and holes were punched into the PDMS. After plasma cleaning, this PDMS block was bonded on top of the thin PDMS film on the master wafer, containing the lower channels. Then, the thin layer was cut along the edges of the PDMS block and the two bonded layers were carefully removed from the wafer. After this, holes for the lower channel were punched into the PDMS. The structured side of the device and a cover slip were plasma cleaned and bonded together as described. The assembled microfluidic through-flow actuator is displayed in Figure 4C. A suspension of *D. discoideum *LimE-GFP cells was filled into the lower channel. After 15 minutes, PB was supplied with a constant flow rate of 5 *μ*l/h for the entire experiment. The pressure in the actuation channel was controlled by a metering valve, located between the channel inlet and the oxygen supply (0 to 1 bar above atmosphere) as shown in Figure 4D. The pressure was measured with a glycerin filled manometer (0 to 1 bar, Swagelok).

## 3. Results

### 3.1. Overlay techniques

**Figure 5:**
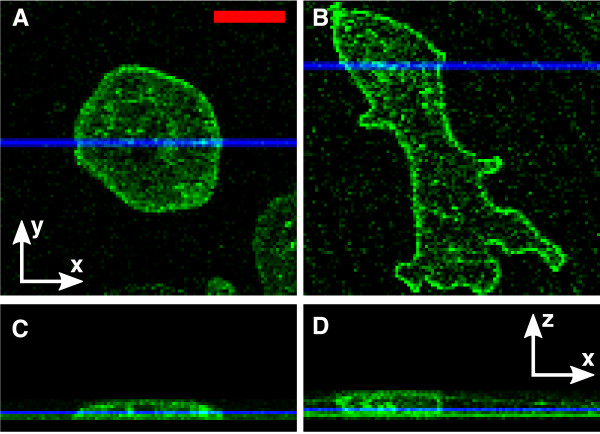
**Flattening of *D. discoideum *under agar and oil overlayer**. cAR1-GFP labeled cells below an agar (A, C) and oil (B, D) overlayer. (A) and (B) show confocal x-y scans. (C) and (D) show the corresponding z-stacks of those cells. The blue lines in (A, B) show the y-values of sections (C, D), and the blue lines in (C, D) show the z-value of images (A, B). Scale bar: 10 *μ*m.

Following the protocol presented above, we flattened *D. discoideum *cAR1-GFP cells by an agar and an oil overlay. In the agar overlay method, the blotting of the fluid between the agar sheet and glass is difficult to control, and demands great care. Under the agar overlay it was possible to flatten the cells to less than 3 *μ*m in height (Figure 5A and 5C). The temporal stability of flattening is limited by the drying of the agar. To overcome this limitation, a humid environment is required, as reported in [18].

**Figure 6:**
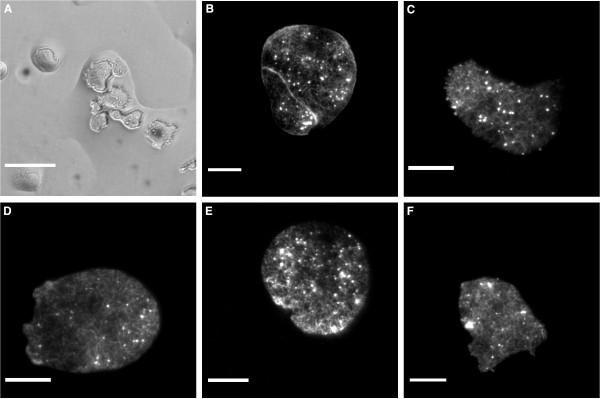
**Sample Images using *D. discoideum *LimE-GFP**. (A) BF image of cells under an oil overlayer. The breakup of the wetting layer can be seen, with flatten cells confined inside small aqueous islands. (B-F) TIRF microscopy images of cells flattened by the agar overlay (B), the oil overlay (C), the single layer microfluidic device (D), the closed-end actuator (E) and the through-flow actuator (F). Scale bar in A is 50 *μ*m and the scale bars in B to E display 10 *μ*m.

An exemplary *D. discoideum *cAR1-GFP cell, flattened to ~3 *μ*m under an oil overlayer is shown in Figure 5B and 5D. We observed that the degree of flattening was prone to strong variations. In particular, flattening was strongly dependent on the distance between the cell and the wick. Flattening was further limited by the breakup of the aqueous layer, see Figure 6A. This break up of the wetting layer into isolated islands was frequently observed when liquid in the aqueous layer was reduced beyond a critical amount. Due to these limitations, the degree of flattening was harder to control, when compared to the agar overlay.

### 3.2. Single layer microfluidic device

**Figure 7:**
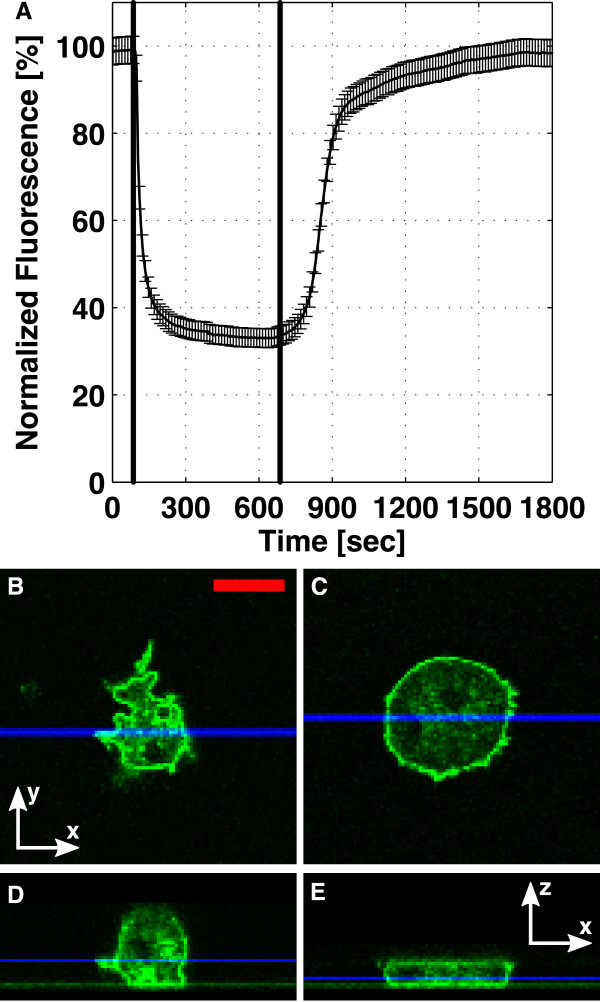
**Characterization of the single layer microfluidic device**. (A) The channel was filled with 100 *μ*M fluorescein, and the intensity in the middle of the device was recorded. Between t = 85 s (line 1) and t = 685 s (line 2), the syringe pump withdrew 250 *μ*l/h of the fluorescein through the device, leading to a drop in the channel height. This flow rate was used to flatten cAR1-GFP cells. Confocal x-y scans are shown for a cAR1-GFP cell (B) before and (C) during flattening. (D, E) show the corresponding x-z sections. The blue lines in (B, C) show the y-values for the sections (D, E), and the blue lines in (D, E) show the z-value of images (B, C). Scale bar: 10 *μ*m.

By adjusting the rate at which fluid is withdrawn from the single layer device, we can tune the degree of flattening. When we fix the flow rate, the channel height will eventually approach a constant value, which in turn allows us to conduct stable, long term microscopy on flattened cells. The device characterization with fluorescein is shown in Figure 7A. Here, we applied a suction speed of 250 *μ*l/h, which reduced the channel height by 67% (from 14 to approximately 4.7 *μ*m). After the fluid flow was stopped, the ceiling relaxed back into its initial position. During the first 5 minutes, 80% of the original height was recovered, but it took more than 16 minutes to relax back into its original height.

The withdrawal speed of 250 *μ*l/h was also used to flatten cAR1-GFP cells. The confocal x-y scans of a cell before flattening, and during flattening are shown in Figure 7B and 7C. The height of the cell decreased from 11 *μ*m (Figure 7D) to ~4 *μ*m (Figure 7E) in this device.

### 3.3. Double layer microfluidic devices

**Figure 8:**
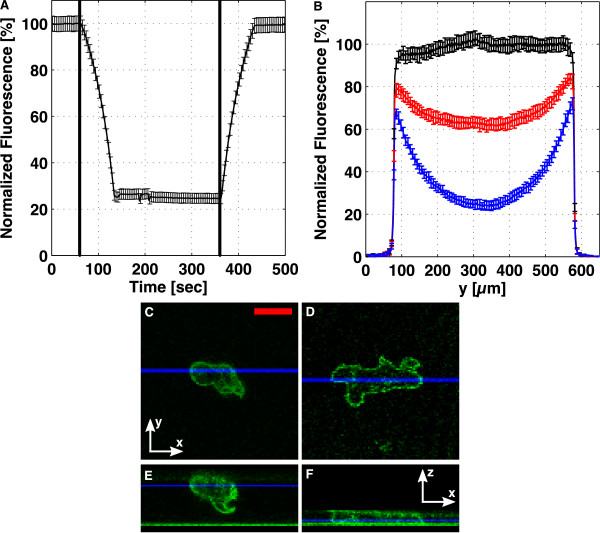
**Characterization of the closed-end actuator**. The lower channel was filled with 100 *μ*M fluorescein, and in (A), the fluorescence in the middle of the channel is displayed, as the pressure in the actuation channel is increased, t = 60 s to t = 130 s, and then, as the pressure is released, t = 360 s to 430 s. (B) shows the profile across the channel for different degrees of flattening, with the black, red and blue curves corresponding to t = 20 s, 110 s, and 300 s in (A). Confocal x-y scans are shown for a cAR1-GFP cell (C) before and (D) during flattening. (E, F) show the corresponding x-z sections. The blue lines in (C, D) show the y-values for the sections (E, F), and the blue lines in (E, F) show the z-value of images (C, D). Scale bar: 10 *μ*m.

**Figure 9:**
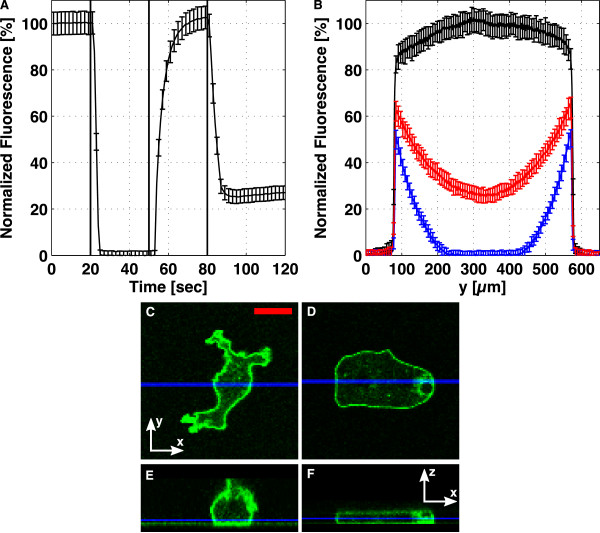
**Characterization of the through-flow actuator**. The lower channel was filled with 100 *μ*M fluorescein, and in (A), the fluorescence in the middle of the channel is shown. At t = 20 s (line 1), the pressure in the actuation channel was increased by 0.4 bar, at t = 40 s (line 2), the pressure was released, and at t = 80 s (line 3), the pressure was increased to 0.2 bar above atmosphere. (B) shows the profile across the channel for different degrees of flattening, with the black, blue, and red curves corresponding to t = 15 s, 40 s, and 105 s in (A). Confocal x-y scans are shown for a cAR1-GFP cell (C) before and (D) during flattening. (E, F) show the corresponding x-z sections. The blue lines in (C, D) show the y-values for the sections (E, F), and the blue lines in (E, F) show the z-value of images (C, D). Scale bar: 10 *μ*m.

In the double layer devices, we tune the height of the cell-containing channel by adjusting the pressure in an actuation channel above. The microfluidic closed-end actuator was characterized in Figure 8. The through-flow actuator was characterized in Figure 9. In these figures, subfigure A shows the temporal evolution of the lower channel height when flattened and unflattened. Subfigure B shows height profiles across the channel, for different degrees of flattening. Subfigures C-F show confocal images for a cAR1-GFP cell before (C, E), and during (D, F) flattening.

For the closed-end device, we saw that increasing the pressure in the actuation channel leads to a decrease of height of the lower channel from 15 *μ*m to ~4 *μ*m (Figure 8A) within approximately 70 seconds. The rates of pressurization and de-pressurization in the actuation channel are governed by the speeds at which the connected syringe pump runs. By running the syringe pump at the same rate, in reverse, we unflattened the lower channel in about the same amount of time it took to flatten. By using higher flow rates on the actuation syringe pump, or by decreasing the initial size of the air bubble in the actuation syringe (see the methods section above), we can decrease the time scales.

In the through-flow device, the time for the lower channel to flatten was even shorter. By quickly adjusting the oxygen pressure in the actuation channel, we were able to change the channel height from 24 *μ*m to less than 1 *μ*m in less than 10 s (Figure 9A). The reverse process had slightly longer time resolution, ~20 s. The limitations in speed and stability of this device are caused by the pressure supply. Pressure is applied by manually opening a needle valve on the supply end, and is released by closing this valve and opening an exhaust needle valve (Figure 4D). Replacing this system with a more sophisticated automated pressure control should improve the stability of the flattened state, and the speed at which flattening/unflattening occurs.

### 3.4. Application to TIRF microscopy

The confocal x-y scans of cAR1-GFP cells before and during flattening show a change from a rather irregular, ruffled geometry to a regular one, where the membrane is firmly pressed against the channel walls. This restriction improves TIRF imaging, since only the surface next to the glass can be visualized. A representative TIRF image of *D. discoideum *LimE-GFP cells for each method is shown in Figure 6B-F. The LimE-GFP label has been established as marker for filamentous actin [19]. Previously, the actin dynamics in these cells was investigated for cells flattened by an agar overlayer. For each of the setups, the actin bundles are visible, implying that the cells have been confined to the glass surface by the individual techniques. Furthermore, in the supplementary material, we show a time lapse TIRF movie of cells in the single layer device (additional file 1).

## 4. Discussion

We have presented classical overlay techniques and more elaborate microfluidic methods for flattening eukaryotic cells. Overlay techniques achieve cell flattening by the removal of the fluid between the glass floor and a confining top layer. The microfluidic approaches rely on applying a pressure difference across a deformable PDMS layer, which acts as a ceiling for the cell flattening chamber. The height of this PDMS ceiling is adjusted by changing this pressure difference.

We used the agar overlay technique as the reference to compare the stability, the degree of control, and the ease of operation for the various cell flattening techniques. The protocol is well known and tested. It has been used, for example, to apply mechanical stress to living cells [20] or to enhance live cell imaging by deforming cells into a quasi two-dimensional geometry [21]. Also, agar overlay experiments have been performed where liquids have been added and removed during flattening [13]. However, the removal and reintroduction of fluid underneath the agar overlay is difficult to perform and hard to control quantitatively, leaving a high risk of damaging the cells.

Variations of the oil overlay method have been used, for example, for the observation of quasi two-dimensional slugs of *D. discoideum *during its developmental cycle [22], as well as to investigate meiosis in insect spermatocytes [23]. The oil overlay technique is harder to control than the agar overlay method. The height of the aqueous layer is dependent on the distance from the wick and suffers from variations due to the breakup of the wetting layer. Furthermore, controlled exchange of the aqueous medium is even more difficult to achieve than in the case of the agar overlay once the cells are confined underneath the oil layer. The oil overlay method also has a number of advantages compared to the agar overlay. For instance, if stable operating conditions are required, the oil overlay is preferable to the agar assay since the latter requires a humid environment to prevent it from drying. For a brief review of overlay techniques see also [23].

To complement the overlay techniques, we have developed single and double layer microfluidic devices to perform well-controlled flattening of living cells. The single layer device is the easiest to fabricate. The high aspect ratio, which is necessary for the operation of this device, allows many cells to be flattened at once. The relaxation time of this device, after flattening, is greater than 15 min. Therefore, this device has only limited applicability for repeated cycles of flattening and relaxing. Furthermore, it is often desirable to decouple the control of flattening from the fluid flow in the channel containing the cells. To accomplish this, an actuation channel is added above the cell flattening channel. In the first variant of this double layer device, the actuator is a closed-end channel, in the second variant, a through-flow channel.

The closed-end actuator is well-suited for long term microscopic imaging experiments, because no continuous flow in the actuation channel is necessary. On the other hand, the through-flow device allows the continuous delivery of oxygen to the cells through the permeable PDMS film between the upper and lower layers. When the pressure is released, these devices recover to their initial states within a minute (see Figures 8A and 9A). Note that the use of spacers to control the thickness of the PDMS film between the actuation and the compression channel makes our protocol less demanding in terms of equipment, when compared to the PDMS spin-coating approach that is commonly used for two-layer microfluidic fabrication [16,24].

The main advantage of microfluidic methods compared to overlay techniques is the high level of control. Flattening can be tuned, and ranges from gentle cell compression, to immobilization by mechanical trapping, and destruction by bursting the cell membrane. Due to the continuous flow, the chemical environment of the cells can be much better controlled inside a microfluidic flattening device. On the one hand, the medium surrounding the cells is continuously exchanged, so that waste products and signaling substances released by the cells themselves are removed and a stationary chemical environment can be implemented. On the other hand, additional chemical factors may be added to the fluid flow and even modulated in the course of the experiment, so that flattening and chemical stimulation can be realized simultaneously. Note that additional valves may be required to add chemical species to the fluid flow upstream of the actual flattening chamber.

Despite these advantages, microfluidic techniques also have a number of drawbacks. Due to the rigidity of PDMS, the ceiling of the microfluidic flattening chambers will not adjust to the shape of the cells. In contrast, the overlay techniques will adapt to some extent to the morphology of the cells (Figure 5C and 5D). This can be advantageous if, for example, large mechanical stress on the nucleus has to be avoided. Besides, the manufacturing of microfluidic devices requires elaborate micro-fabrication techniques like photolithography and polymer molding. Consequently, the assembly of microfluidic devices is more time consuming than the overlay techniques. Usually it takes several days to setup a microfluidic device, beginning with the chrome mask generation. The operation of microfluidic setups is also considerably more expensive, since advanced tools like micro-liter syringes and precision pumps are required. Great care has to be taken to avoid leakage or inclusions of air in the tubing or the micro-chamber. Such gas inclusions would obstruct the stable operation of a microfluidic device because of their large compressibility. This is especially true for the single layer microfluidic device, which achieves flattening by withdrawing the liquid from the channel.

## 5. Conclusions

For imaging experiments, which do not require the application of drugs or a constant supply of nutrients, the agar overlay method is the most reliable. If a continuous fluid flow is needed, or direct and precise control of flattening is desired, then the microfluidic based flattening devices are superior to the overlay techniques. Among the microfluidic cell flattening devices described here, the through-flow actuator combines the advantages of high controllability, medium construction time, and reproducibility. To conclude, we summarize the key advantages and disadvantages of the five setups in Table 1.

**Table 1:** Comparison of the cell flattening devices

Name	Control of Flattening	Control of Environment	Construction Time
Agar overlay	no direct control^1^	no	< 1 hour
Oil overlay	no direct control^1^	no	< 1 hour
Single layer	via syringe pump	yes^2^	2-3 hours^3^
Closed-end actuator	via actuation channel	yes (lower channel)	> 3 hours^3^
Through-flow actuator	via actuation channel	yes (lower channel)	2-3 hours^3^

Comparison of devices for eukaryotic cell flattening. Remarks: ^1^Indirect control via the removal of liquid below the overlayer. ^2^The syringe pump by which the collapse of the ceiling is controlled, might also be used to suck in nutrients or drugs. ^3^The time estimate takes only the assembling of microfluidic channels into account. The chrome mask generation, patterning of the Si-wafer, and the following molding of the PDMS take more than a day.

## Supplementary Material

Additional file 1 **Time lapse TIRF-microscopy movie of *Dictyostelium discoideum *LimE-GFP during flattening**. A *Dictyostelium discoideum *LimE-GFP cell is flattened with a single layer device. The LimE-GFP labels specifically F-actin. The images were taken at a rate of 2 per second and are displayed at 10 frames per second. The movie was subject to binary thresholding using MATLAB. Click here for file
